# 159 Nurse–Patient Relationships in Airborne Infection Isolation Rooms: A Qualitative Study Based on the Fundamentals of Care Framework

**DOI:** 10.1017/ash.2026.10560

**Published:** 2026-06-23

**Authors:** Kira Elizondo, Jose Cadena Zuluaga, Gaielle Harb

**Affiliations:** 1 University of Texas Health Science Center at San Antonio; 2 University Of Texas Health Science Center at San Antonio, South Texas Vetrans Healthcare System

## Abstract

**Background:** Central-line associated bloodstream infections (CLABSI) are defined as bacteremia, or fungemia, in patients with a central intravascular device and no other apparent source of infection. In cases where devices are not removed, treatment of CLABSI involves eradication of intraluminal infection with systemic antibiotics and supratherapeutic concentrations of antibiotics inside the catheter, creating an antibiotic lock. Antibiotic locks can also be used in selected high-risk individuals and those with recurrent CLABSI for prevention. In this review, we evaluated the use of antibiotic locks for the treatment and prevention of CLABSI at South Texas Veterans Health Care System (STVHCS) to assess if use has been in accordance with standard of care. Methods Retrospective data review of STVHCS electronic medical records from 2020-2025. Data collected is limited to age, catheter type, purpose of antibiotic lock (treatment or prevention), organism targeted, antibiotic used for lock, duration, use of systemic antibiotics, and if the intervention was successful at 30 and 90 days. Appropriate lock used was defined as in accordance with the IDSA 2009 CLABSI treatment guidelines, or the 2022 SHEA compendium. Results Data available demonstrated that over the past 5 years there have been 18 instances of antibiotic lock use in 16 patients. Of this population, 13 of 18 lines involved were tunneled hemodialysis catheters with the remaining being ports (3), and tunneled central lines (2). Six of these instances were used for prevention of CLABSI due to long-term hemodialysis catheters/recurrent CLABSI, while the remaining 12 were used as treatment. Per IDSA CLABSI guidelines, removal of a catheter was recommended for 8 of 18 cases, (Staphylococcus aureus, Enterococcus spp., and metastatic infection). Of those cases, 6 patients had removal of catheter, whereas the remaining 2 patients had catheter salvage. The retention of the catheters were in accordance with IDSA guidelines as they received systemic antibiotics with concomitant lock therapy and had no alternative venous access and removal was contraindicated. We found at 30 days, all patients had survived. Whereas at 90 days, 4 patients had expired due to other all-cause mortality and unrelated to recent CLABSI infection. Conclusions Based on this retrospective review, there were 18 instances of antibiotic lock therapy that appeared to be in accordance with clinical guidelines for both prevention and treatment of CLABSI. This data review is part of the antibiotic stewardship program and will be used to develop future quality improvement processes.

